# Biomechanical analysis of a new cannulated screw for unstable femoral neck fractures

**DOI:** 10.3389/fbioe.2024.1382845

**Published:** 2024-05-13

**Authors:** Zhigang Chen, Feiyang Chen, Xinbao Xu, Xin Li, Haidong Cui, Wen Zhang, Dong Jiang, Feixiang Zhang, Yinbing Chen, Shiran Zhou, Shujun Lyu

**Affiliations:** ^1^ Department of Orthopaedics, Hai’an People’s Hospital, Hai’an, Jiangsu, China; ^2^ Department of Orthopaedics, Affiliated Hai’an Hospital of Nantong University, Hai’an, Jiangsu, China; ^3^ Medical School, Yangzhou University, Yangzhou, Jiangsu, China; ^4^ Institute of Orthopaedics, Soochow University, Suzhou, Jiangsu, China

**Keywords:** unstable femoral neck fracture, new cannulated screw, internal fixation, biomechanics, finite element analysis

## Abstract

**Background:**

The treatment of unstable femoral neck fractures (FNFs) remains a challenge. In this study, a new cannulated screw for unstable FNFs was designed to provide a new approach for the clinical treatment of these injuries, and its biomechanical stability was analyzed using finite element analysis and mechanical tests.

**Methods:**

An unstable FNF model was established. An internal fixation model with parallel inverted triangular cannulated screws (CSs) and a configuration with two superior cannulated screws and one inferior new cannulated screw (NCS) were used. The biomechanical properties of the two fixation methods were compared and analyzed by using finite element analysis and mechanical tests.

**Results:**

The NCS model outperformed the CSs model in terms of strain and stress distribution in computer-simulated reconstruction of the inverted triangular cannulated screw fixation model for unstable FNFs. In the biomechanical test, the NCS group showed significantly smaller average femoral deformation (1.08 ± 0.15 mm vs. 1.50 ± 0.37 mm) and fracture line displacement (1.43 ± 0.30 mm vs. 2.01 ± 0.47 mm). In the NCS group, the mean stiffness was significantly higher than that in the CSs group (729.37 ± 82.20 N/mm vs. 544.83 ± 116.07 N/mm), and the mean compression distance was significantly lower than that in the CSs group (2.87 ± 0.30 mm vs. 4.04 ± 1.09 mm).

**Conclusion:**

The NCS combined with two ordinary cannulated screws in an inverted triangle structure to fix unstable FNFs can provide better biomechanical stability than CSs and exhibit a length- and angle-stable construct to prevent significant femoral neck shortening.

## 1 Introduction

Femoral neck fractures are relatively common hip fractures, of which 3% are aged ≤50 years, mostly due to high-energy injuries (Robinson et al., 1995). For younger patients, internal fixation is still the best treatment (Duffin and Pilson, 2019), but this treatment is difficult to apply because of the high shear stress, poor stability, and postoperative complications such as fracture redisplacement, fracture nonunion, femoral neck shortening and ischaemic necrosis of the femoral head involved in Pauwels type III FNFs (Xu et al., 2019). Early anatomic reduction and effective internal fixation affect the treatment outcomes (Barnes et al., 1976).

Various methods of fixation have been used previously for these injuries, such as cannulated screws, dynamic hip screws, proximal locking plate fixation system (Mubark et al., 2021), femoral neck system (Mehraj et al., 2022), et al. Currently, a consensus regarding of the surgical implant for these injuries is still lacking, and considerable controversy remains in the literature and among surgeons (Merz et al., 2015; Slobogean et al., 2015). Three parallel cannulated screws represent a commonly used internal fixation device for challenging fractures (Swart et al., 2017). CSs have the advantages of small incision, less intraoperative bleeding, short operation time, reliable fixation and favorable fracture healing while maintaining the biological environment of the fracture site. However, the stability of the fracture site is poor, and screw withdrawal often occurs, leading to internal fixation failure (Parker, 2009; Yang et al., 2013). Therefore, some methods have been recommended to improve stability, such as changing the configuration and number of screws (Jiang et al., 2021a), using the off-axis screw technique (Jiang et al., 2021b), combining a medial femoral support plate (Wang et al., 2023), and utilizing a posterior fully threaded positioning screw (Shin et al., 2020), double-head cannulated screws (DhCSs) (Zhang et al., 2020). Dynamic hip screws (DHS) are another common fixation method. DHS has angular stability, and a plate fixed on the lateral cortex can provide reliable support for fractures with severe posterior comminution of the femoral neck, but there are corresponding shortcomings, such as high surgical trauma, poor antirotation ability, and high cost (Stoffel et al., 2017), Some scholars have demonstrated that three peripherally inverted triangle CSs within the femoral neck were biomechanically better than a more centrally fixed DHS construct with an anti-rotation screw in axial and torsional stability in the repair of unstable FNFs (Wright et al., 2020). Kemker et al. reported that a DHS with a derotation screw was biomechanically similar to an inverted triad of CSs in these injuries (Kemker et al., 2017). In recent years, the medial support of the femoral neck has received increasing attention in the treatment of femoral neck fractures (Liu et al., 2019). Wright et al. reported that posterior inferior comminution significantly affected torque to failure in unstably oriented FNFs (Wright et al., 2020).

Taking the above factors into consideration, we hypothesized that replacing the inferior cannulated screw with a newly designed cannulated screw may improve stability through the nut fixed on the lateral cortex, maintain the length of the femoral neck, and realize immediate static fracture compression intraoperatively, while allowing fracture compression in the superior region postoperatively. Therefore, we compared the biomechanical stability of the NCS with that of the CSs through finite element analysis and biomechanical experiments to provide a theoretical basis for the application of the NCS in clinical practice.

## 2 Methods

### 2.1 Development of a new cannulated screw

**FIGURE 1:**
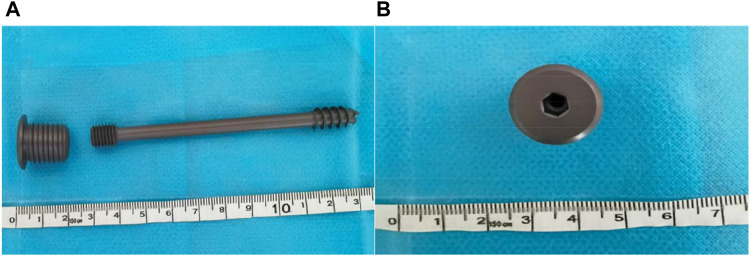
The new cannulated screw. **(A)** Anterior-Posterior (AP) view of the NCS. **(B)** Lateral view of the nut.

**FIGURE 2:**
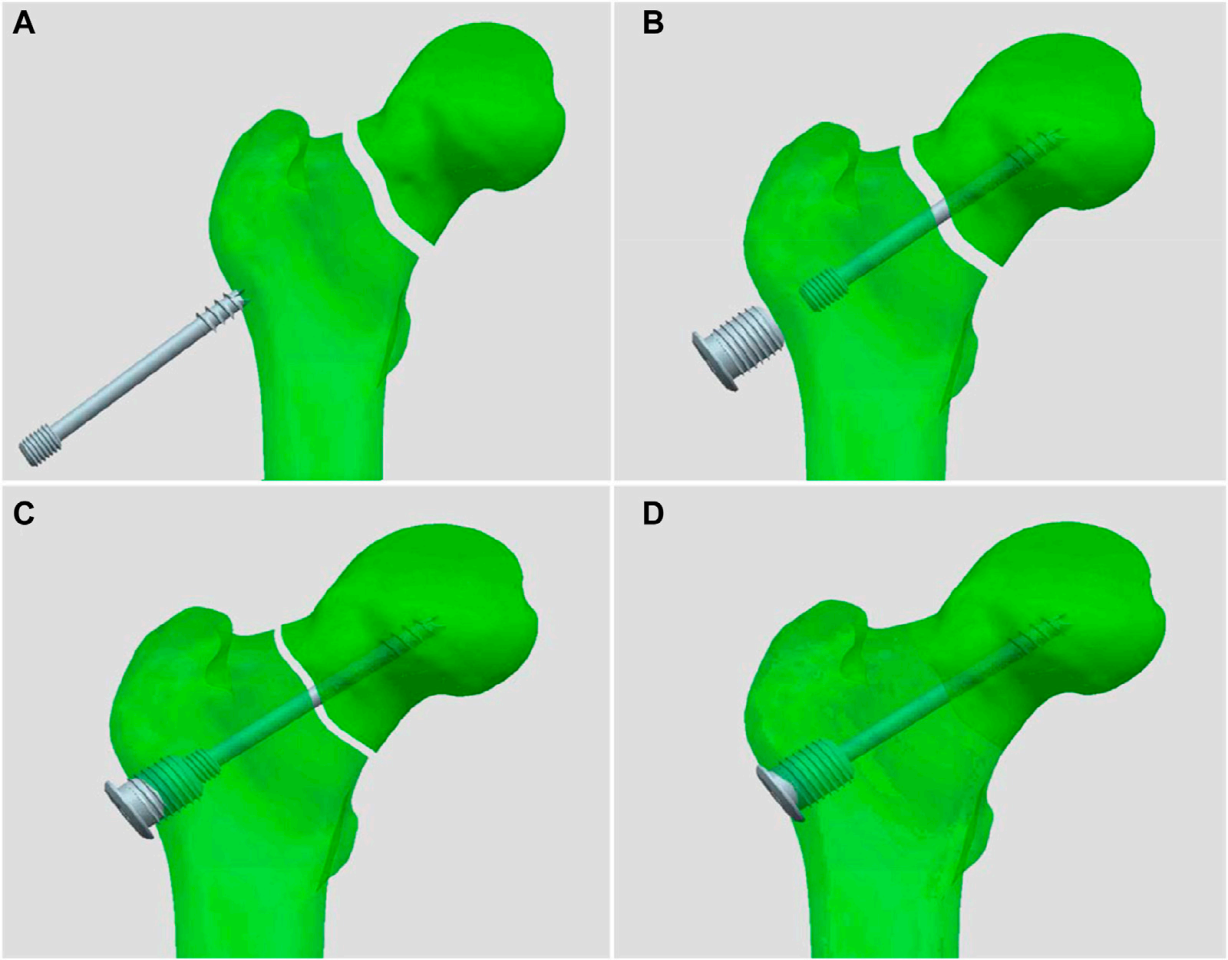
Schematic diagram of compression and fixation of the new cannulated screw. **(A)** The screw inserting, **(B)** The nut inserting, **(C)** Compression beginning, **(D)** The length- and angle-stable construct forming.

Our new designed cannulated screw (Chinese utility model patent, No. ZL201720445692. X) consists of two parts: the proximal end with 7.3 mm half-threads which is the same as those ordinarily used in clinical practice, and the distal end of the screw is modified to have 5 mm diameter threads, and a matching nut with internal threads can be fixed with the distal end of the screw (Figure 1). The process of engaging the distal end of the screw with the nut enables static compression of the fracture end, avoiding repeated cutting of cancellous bone by the proximal threads during compression with ordinary screws. At the same time, the nut also has external threads on the outer side, which can be fixed on the lateral cortex, preventing significant femoral neck shortening and screw withdrawal. Different nut lengths were chosen to achieve different degrees of compression. The diameter of the external threads of the nut was 10 mm, which is smaller than that of the DHS and can be locked with the proximal lateral cortex to form a length- and angle-stable construct, and the whole device achieves an integrated frame fixation of the femoral head and the femur, which can obtain femoral head anti-rotation (Figure 2).

### 2.2 Finite element analysis

**FIGURE 3:**
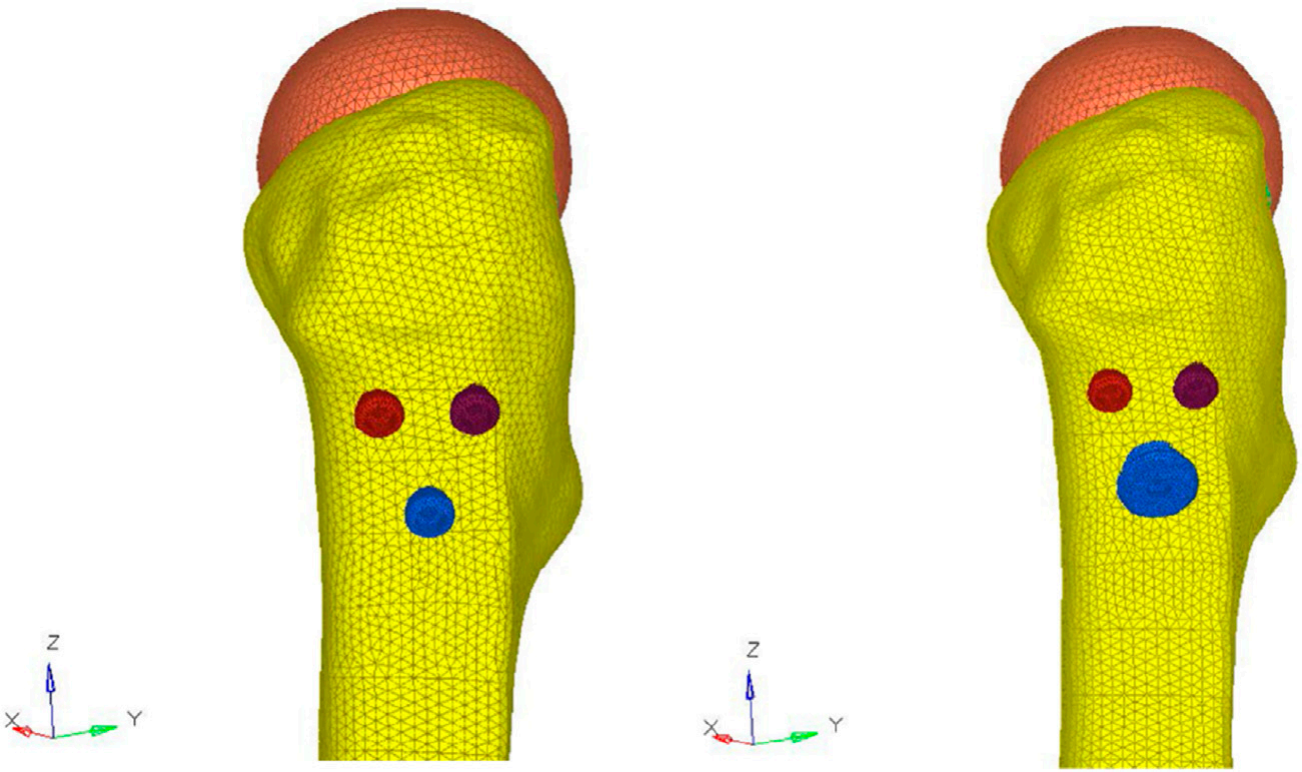
Number of nodes and elements for both fixation models. CSs model: 412942 nodes, 260183 elements. NCS model: 442846 nodes, 277052 elements.

This study was approved by the Ethics Committees of Hai’an People’s Hospital and Affiliated Hai’an Hospital of Nantong University (reference number HKL2016042). A 33-year-old healthy young male volunteer signed an informed consent form, excluding chronic diseases, pelvic and lower extremity deformities, fractures and a surgical history. A 64-slice CT scanner (Siemens Company, Germany) was used to continuously scan the left lower extremity, and the 2D CT image data with a scanning layer of 0.8 mm were stored in.dicom format. The.dicom format file was imported into Mimics 15.0 medical image processing software (Materialise Company, Belgium) to create a 3D model of the full-length femur. The above model was imported into Geomagic Studio 12.0 (Raindrop Company, United States of America) in.stl format to complete the surface model, which was then imported into the ProEngineer 5.0 software (PTC, United States of America) to create a 3D solid model; the cannulated screw model was also established in ProEngineer 5.0 software. The cannulated screw model and femoral model were grouped as required for Boolean operation, and the assembly of each model was completed. The assembled model was imported into Hypermesh 12.0 software (Altair Company, United States of America), and each part of the model was created as a component. For the subsequent loading analysis, an osteotomy model was established with the distal femoral osteotomy plane 10 cm above the femoral condyle. The femoral neck was cut at an angle of 70° from the vertical line of the long axis of the femur to simulate a femoral neck fracture, with a fracture gap of 0.2 mm (Zhao et al., 2021). The femur model was analyzed and divided into four parts: the cortical bone, cancellous bone, femoral neck, and femoral head. After quick edit processing of each part, the body mesh was divided, and a tetrahedral Solid187 cell mesh was chosen. The numbers of nodes and elements of both fixation models are shown in Figure 3, and the elastic moduli of the bone and implants are listed in Table 1. Boundary constraints were applied to the model. Vertical forces of 600 N (simulating the standing of one leg), 1000 N, 1400 N (equivalent to 2 times the body weight of the patient) and 2100 N (equivalent to 3 times the body weight of the patient) were applied to the femoral head (Eberle et al., 2011; Pagnano et al., 2011; Kemker et al., 2017). The stability of the two internal fixation methods was analysed using Ansys13.0 software (ANSYS company, United States of America).

**TABLE 1:** Bone and internal fixation material properties.

Material	Elastic modulus (MPa)	Poisson’s ratio
Cortical bone	16800.0	0.3
Cancellous bone	840.0	0.3
Femoral head	900.0	0.29
Femoral neck	620.0	0.29
Titanium alloy	110000.0	0.3

### 2.3 Biomechanical testing

A study showed that the biomechanical properties of fourth-generation composite femurs under bending, axial and torsional loading were similar to those of cadaveric specimens, with less variability than that in cadaveric specimens (Elfar et al., 2014). Referring to the efficacy analysis of Stoffel et al., 2017 (Stoffel et al., 2017), we selected 10 Sawbone femurs (left, fourth generation, Pacific Research Laboratories, Washington, United States of America) to mimic a Pauwels type III femoral neck fracture. A vertically oriented osteotomy 70° from the horizontal plane was performed by the same orthopaedic surgeon, the specimens were then divided into two groups of five. In the CSs group, three partially threaded cannulated screws (Titanium alloy, 7.3 mm, Jiangsu Adiel Medical Technology Co., China) were used to fix the femoral head in parallel with the standard inverted triangle, while in the NCS group, two superior partially threaded cannulated screws (Titanium alloy, 7.3 mm, Jiangsu Adiel Medical Technology Co., China) and one inferior new cannulated screw (Titanium alloy, 7.3 mm, Jiangsu Adiel Medical Technology Co., China) were used to fix the femoral head in the same way.

**FIGURE 4:**
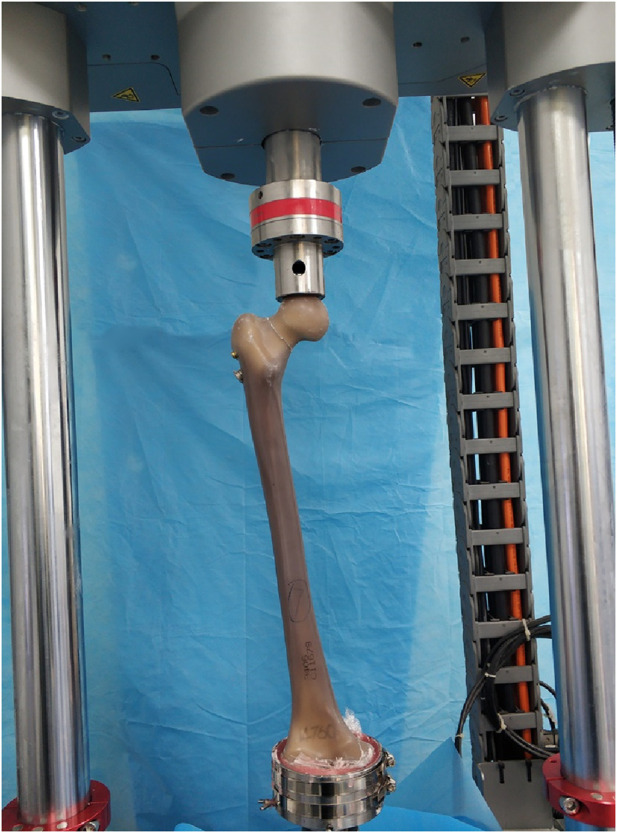
During the biomechanical tests, the femur was exactly in the vertical position and the femoral shaft was angled by 11° from the force line.

Biomechanical testing was performed using a tensile-torsional biaxial universal mechanical testing machine (Instron E10000, Instron Corporation Norwood, MA, United States of America). The distal femur was placed in a custom-made jig supported by dental brace powder, and the femur was angled 11° from the force line (Cristofolini et al., 1996). The femoral head was placed directly in a special fixture on a mechanical testing machine. The distance between the upper and lower indenters was adjusted so that the femur was exactly in the vertical position and the femoral shaft was angled by 11° from the force line (Figure 4). A cyclic test was first performed with a load of 0–1000 N at a frequency of 2 Hz for 2000 cycles, and the change in the displacement of the fracture break and femur was measured at the end of the fatigue test. After the cyclic test, vertical compression was performed at a rate of 3 mm/min until the load reached 1400 N and 2100 N (approximately equal to 3 times the body weight of an adult weighing 70 kg) respectively (Kemker et al., 2017). The load-displacement curves were recorded, and the stiffness was calculated to compare the fixation effect between the two models.

### 2.4 Statistical treatment

Statistical analysis was performed using SPSS software (version 26.0; IBM, United States of America), and data were expressed as the mean and standard deviation of continuous variables, Femoral deformation, fracture line displacement, and stiffness of the two groups were compared using an independent sample *t*-test. Statistical significance was set at *p* < 0.05.

## 3 Results

### 3.1 Deformation and von mises stress distribution of the two groups of internal fixation screws

**FIGURE 5:**
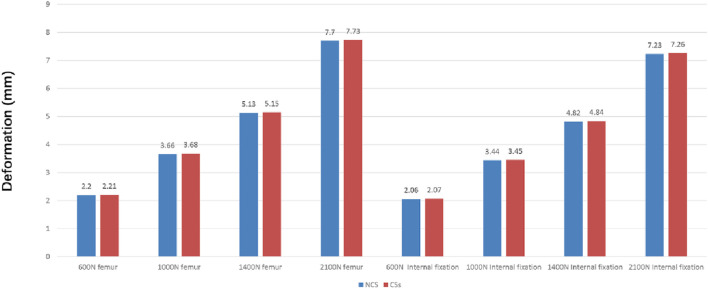
The maximum deformation of the two group under vertical loads of 600, 1,000, 1,400, and 2,100 N.

**FIGURE 6:**
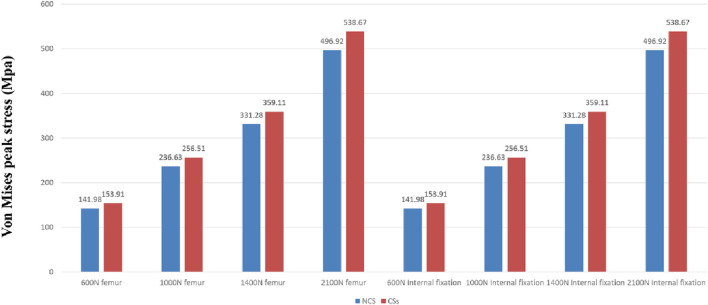
The peak stress of the two groups under vertical loads of 600, 1,000, 1,400 and 2,100 N.

**FIGURE 7:**
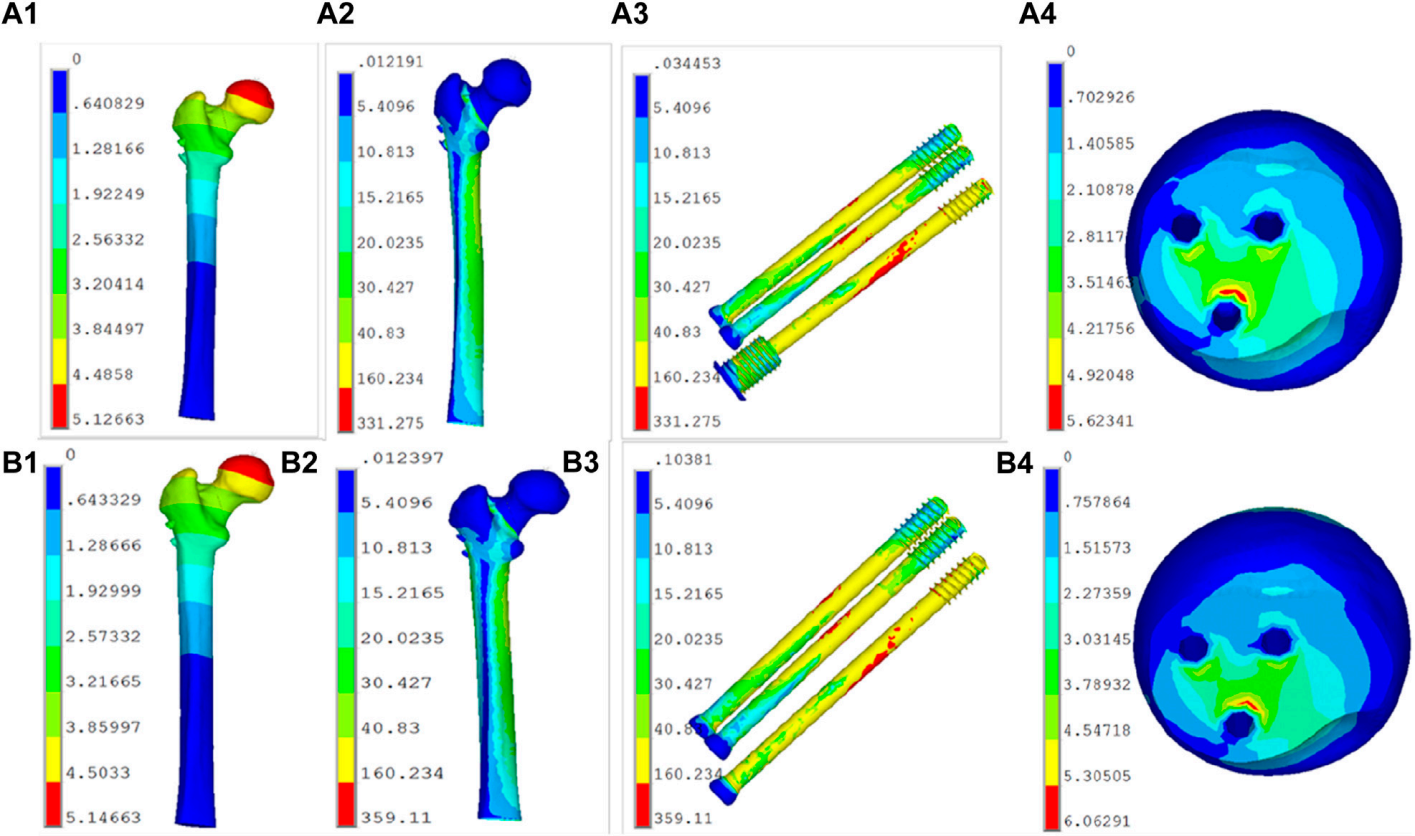
The deformation and stress distribution on the two groups. (**A1,A2,A3,A4**): the NCS model; **(B1,B2,B3,B4)**: the CSs model.

The deformation nephograms of the two groups showed that the maximum deformation of the NCS group under vertical loads of 600 N, 1000 N, 1400N and 2100 N was slightly lower than that of the CSs group (Figures 5, 7), and that the peak stress of the NCS group under vertical loads of 600, 1,000, 1400N and 2100 N was significantly lower than that of the CSs group (Figures 6, 7). The peak stress of the two groups appeared in the middle of the inferior screw, which was close to the fracture line (Figure 7).

### 3.2 Biomechanical test results of both groups

The femoral head was loaded to 0–1000 N to complete the fatigue test and then loaded to 1400 N to complete the static compression test. The biomechanical test results for all specimens are shown in Tables 2, 3. The fatigue test results showed that the femur deformation in the NCS group was 1.08 mm, which was 28% lower than that in the CSs group (1.50 mm). The fracture line displacement in the NCS group was 1.43 mm, which was 28.9% lower than that in the CSs group (2.01). The stiffness of the femur mechanically represents its resistance to deformation. The static test results showed that the femur shortening of the NCS group was 2.87 mm, which was 29% lower than that of the CSs group (4.04 mm), and that the femur stiffness of the NCS group was 729.37 N, which was 33.9% higher than that of the CSs group (544.83 N).

**TABLE 2:** Deformation on the femur model in two groups after fatigue testing.

Specimen	NCS group			CSs group		
	Vertical load (N)	Deformation (mm)	Fracture line displacement (mm)	Vertical load (N)	Deformation (mm)	Fracture line displacement (mm)
1	0–1,000	1.26	1.71	0–1,000	1.79	2.53
2	0–1,000	1.10	1.66	0–1,000	1.93	2.48
3	0–1,000	1.07	1.22	0–1,000	1.35	1.83
4	0–1,000	1.12	1.52	0–1,000	1.02	1.53
5	0–1,000	0.84	1.02	0–1,000	1.39	1.67
Mean (SD)	n/a	1.08(0.15)^a∗^	1.43(0.30)^b∗^	n/a	1.50(0.37)^a^	2.01(0.47)^b^

Student’s t-test: *indicates that the difference is statistically significant.

**TABLE 3:** The stiffness of the femoral models in two groups were compared by static compression test.

Specimen	NCS group			CSs group		
	Vertical load (N)	Compression displacement (mm)	Axial stiffness (N/mm)	Vertical load (N)	Compression displacement (mm)	Axial stiffness (N/mm)
1	2,100	3.17	641.61	2,100	3.91	536.95
2	2,100	2.9	725.05	2,100	5.93	354.39
3	2,100	3.13	670.93	2,100	3.75	560
4	2,100	2.67	756.85	2,100	3.22	653.05
5	2,100	2.46	852.39	2,100	3.39	619.77
Mean (SD)	n/a	2.87(0.30)^a*^	729.37(82.20)^b*^	n/a	4.04(1.09)^a^	544.83(116.07)^b^

Student’s t-test: * indicates that the difference is statistically significant.

## 4 Discussion

Pauwels type III FNFs are challenging to treat and are considered to be “unresolved fractures.” These fractures often occur in young patients because of high-energy injuries, and hip-preserving internal fixation remains the treatment of choice (Su et al., 2021). With the advantages of small incision, less blood loss, less trauma, and shorter hospital stay, cannulated screws remain a more widely used internal fixation method (Sağlam et al., 2014; Xia et al., 2021). Traditional cannulated screws are subject to dynamic pressures and cannot provide enough support for a femoral neck fracture. Under the strong vertical shear force produced by unstable femoral neck fracture, cannulated screws are particularly prone to redisplacement, resulting in complications, such as impaired fracture healing, shortening of the femoral neck deformity, and internal carotid femoral deformities. Zhao et al. demonstrated that the incidence of femoral neck shortening after parallel cannulated screw fixation of Pauwels type II FNFs was 30.33%, which might be related to posterior comminution and poor reduction quality (Zhao et al., 2021). Femoral neck shortening can affect the hip joint function (Zlowodzki et al., 2008). Therefore, the use of three traditional cannulated screws is not recommended for the treatment of unstable femoral neck fractures (Ye et al., 2015). Regarding the treatment of femoral neck fractures, an increasing number of researchers have focused on the integrity and strong support of the medial wall. Huang, et al. demonstrated that CSs combined with medial buttress plate showed less femoral neck shortening than CSs only, but with an additional approach for plating and more blood loss (Huang et al., 2022). Shin et al. used a posterior fully threaded positioning screw to improve fracture healing without further femoral neck shortening (Shin et al., 2020). Similarly, Zhang et al. reported that the use of DhCSs decreased femoral neck shortening (Zhang et al., 2020). Considering that unstable femoral neck fractures require sufficient tension to achieve reduction and stable medial support, we designed a new cannulated screw structure to be placed on the medial inferior side of the femoral neck, combining with two traditional cannulated screws to treat unstable femoral neck fractures. The new cannulated screw structure includes a screw and a nut. The internal thread of the nut was matched to the external thread of the screw tail. When the nut was inserted more in the lateral cortex, the screw tail was extracted more to produce a larger immediate compression than that of the CSs. Moreover, in the process of compression between fracture fragments, the proximal end of the screw and the femoral head were static, thus avoiding the cutting of cancellous bone by the external thread of the partially threaded screw when slightly more compression is needed, which may reduce the holding force of the screw and stiffness of the internal fixation. Furthermore, the screw could form a length- and angle-stable construct to prevent significant femoral neck shortening by locking with the proximal lateral cortex with the external thread of the nut. The diameter of the nut was larger than the distal end of a DhC, which provided greater support to the proximal lateral cortex than a CS and a DhC. Another common complication after cannulated screw fixation for FNFs is screw cut-out. Yueh Wu, et al. found that the incidence of screw cut out was 14.6%, which may be related to elderly patients (age >60 years) and osteoporosis in their study (Wu et al., 2019), so in our future clinical study, we should apply the NCS to young patients with strong bone quality, avoid drills or screws penetrating out of femoral head intraoperatively, and instruct the patients to delay the weight-bearing time postoperatively.

The finite element analysis results of this study showed that the maximum deformation of the NCS group was smaller than that of the CSs group, indicating that the fixation strength and biomechanical properties of the NCS were significantly better than those of the CSs. Additionally, the peak stress of the two groups appeared in the middle part of the inferior screw, close to the fracture line, indicating that the inverted triangle fixation of Pauwels type III FNFs can effectively avoid stress concentration, and it is not prone to failure due to excessive stress concentration in one screw, similar to the results of Merz et al., 2015 (Merz et al., 2015).

We further corroborated the stability of the newly developed cannulated screw using biomechanical experiments with the fourth-generation composite femur model, and the results showed that the femur deformation and fracture line displacement were significantly lower in the NCS group than in the CSs group, while the vertical stiffness was significantly higher than in the CSs group. This also confirmed that the fixation effect of the NCS group was significantly better than that of the CSs group, both in the fatigue test (vertical load 0–1000 N) and in the static test (final vertical load 2100 N, equivalent to 3 times the body weight). This may be related to the caudal nut of the new screw design, which is fixed to the femoral cortex to provide angular stability and maintain the length of the femoral neck, which can improve shear resistance and effectively prevent femoral neck shortening. Moreover, the two superior half-threaded screws still allow slight compression at the fracture site and benefit healing, consistent with the findings of Shin et al., 2020 (Shin et al., 2020).

In this study, the finite element experimental results were corroborated through further biomechanical experiments using a composite femur model, which fully demonstrated the reliability of the experimental results. However, this study has some limitations. First, we did not consider bone density and image gray values as a method of assigning material properties to the model according to related studies (Amirouche et al., 2016). Second, we selected a composite femur model and did not consider the bone distribution of the femoral neck, which was related to the choice of the length of the thread affecting the stress distribution in the femoral neck. Although the corresponding biomechanical properties are similar to those of human cadaveric specimens, there are still differences between them; however, the fracture model is more idealized and ignores the effects of periprosthetic muscles and ligaments on fracture stability. Third, the newly developed screw was not compared with other internal fixation methods, such as DhCSs and femoral neck system (FNS). For further clinical research, the design of the shape, diameter and thread pitches of the new screw caudal nut must be further optimized. Finally, owing to the limited laboratory conditions, only one vertical stress mode was set for mechanical loading. In the actual walking process, the stress mode will change significantly, and A-P bending tests will be carried out in a later experiment for further verification.

## 5 Conclusion

In this study, we designed a new type of cannulated screw to treat unstable femoral neck fractures. Biomechanical testing indicates that NCS with a length- and angle-stable construct for unstable femoral neck fractures can provide better biomechanical stability than CSs and prevent significant femoral neck shortening, providing a new idea for the treatment of young patients with unstable fractures of the femoral neck.

## Data Availability

The raw data supporting the conclusion of this article will be made available by the authors, without undue reservation.
